# Supplementary material for: Antibiotic sensitivity reveals that wall teichoic acids mediate DNA binding during competence in Bacillus subtilis
Source: Nat Commun. 2018 Nov 29;9:5072. doi: 10.1038/s41467-018-07553-8 (PMC6265299; doi:10.1038/s41467-018-07553-8)
Supplement: Supplementary file 1 — Supplementary Information [file 41467_2018_7553_MOESM1_ESM.pdf]

Supplementary Information

Antibiotic sensitivity reveals that wall teichoic acids mediate DNA binding during competence in *Bacillus subtilis*

Mirouze *et al*.

Supplementary Fig. 1

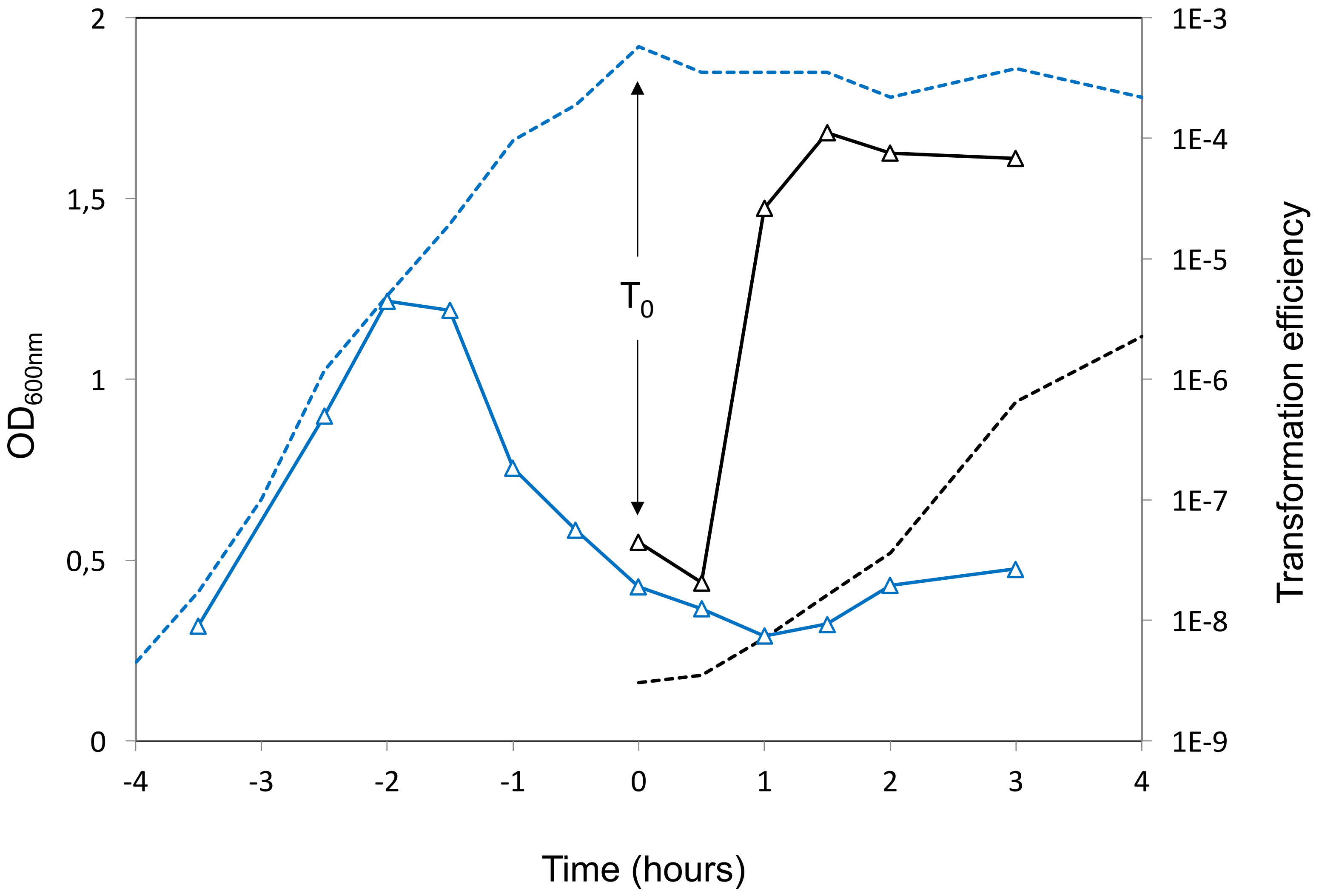

Supplementary Fig. 1. Two-steps transformation protocol.

In this study, we used a two-steps protocol to test the effect of diverse antibiotics and gene deletions on genetic transformation. Our protocol was similar to what was described in [23,24]. Representative curves obtained with the wild-type strain (168) in the absence of antibiotics are shown. Basically, *B. subtilis* strains were first grown in unsupplemented SPI medium (blue dotted line). Upon entry in stationary phase, cultures were diluted 10 times in fresh SPII medium supplemented with antibiotics where indicated (T_0), and growth was allowed to continue for up to 3 hours (black dotted line). Along these two consecutive growth steps, we evaluated every 30 minutes the transformation efficiency (blue and black triangles, respectively). To this end, chromosomal DNA (1μg/ml) harboring an antibiotic resistance cassette was added to an aliquot (1 ml) of the culture for 30 minutes. After 10 minutes of DNase treatment to degrade untransformed DNA, cells were washed once with SPII medium free of drug and then plated on LB plates for viability calculation and on LB plates supplemented with the adequate antibiotic to measure the number of transformants. Transformation efficiencies were calculated by dividing the number of transformants per ml of culture by the viability at each time point. Even though some genetic transformation ($< 10^{-5}$) can be observed in SPI medium, maximum efficiency ($> 10^{-4}$) is reached after approximately 1.5 hours of growth in SPII medium.

a

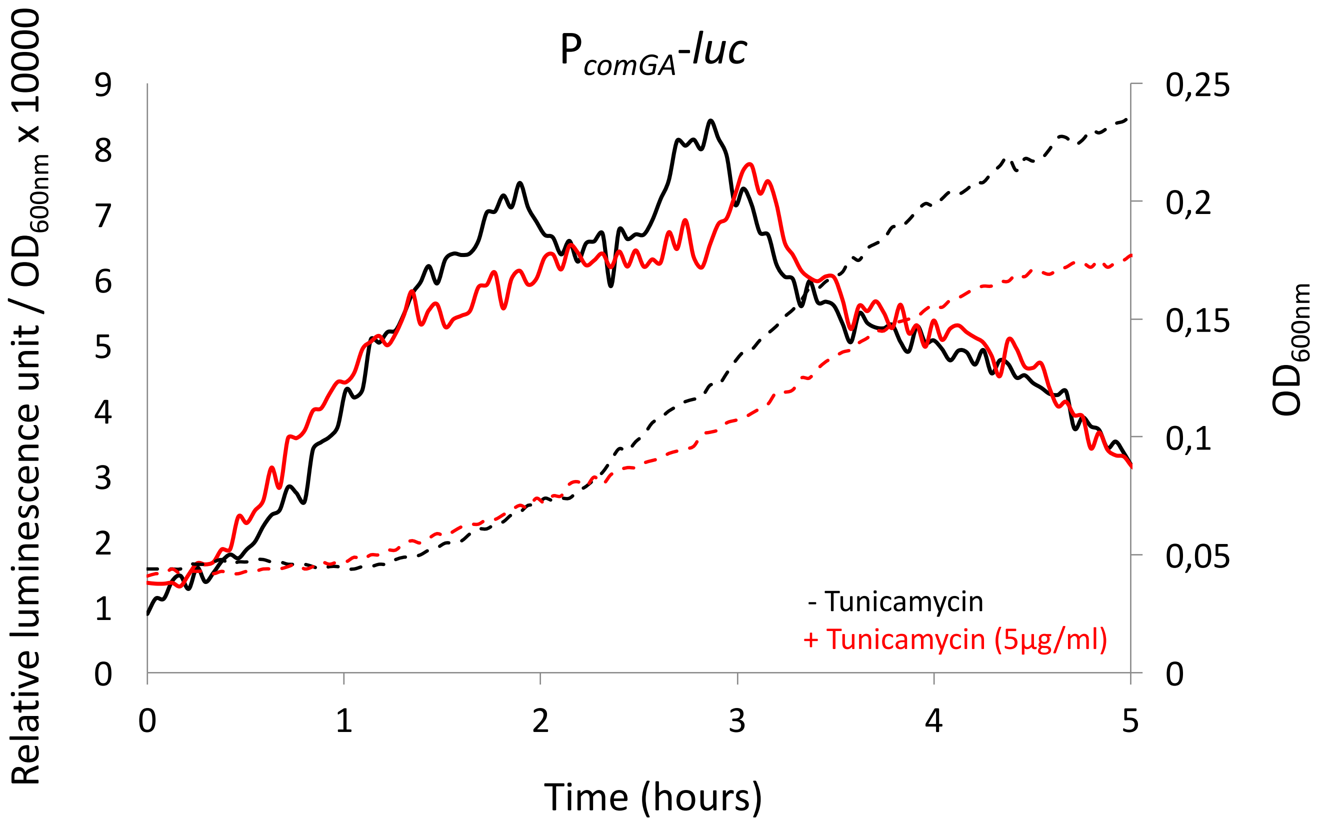

b

	- tuni	+ tuni
% of competent cells (NC59)	5,75% (1,93)	5,57% (1,14)

Supplementary Fig. 2. Tunicamycin does not inhibit competence development.

a. Transcription from the *comGA* promoter fused to the firefly luciferase (strain NC3) in SPII medium. After growth in SPI medium to stationary phase, cells were diluted 10 times in SPII containing (red curve), or not (black curve) tunicamycin (5 μg/ml). Following dilution (T_0), growth (dotted lines, right axis) and transcription rate (plain curves, left axis) were measured every 2 minutes.

b. The percentage of competent cells (strain NC59, expressing *comK-gfp* under the control of the *comK* promoter) was calculated after two hours of growth in SPII medium, in the absence and presence of tunicamycin (5 μg/ml). More than 2000 cells (over 3 experiments) were examined microscopically for each condition. The standard deviation is indicated between brackets.

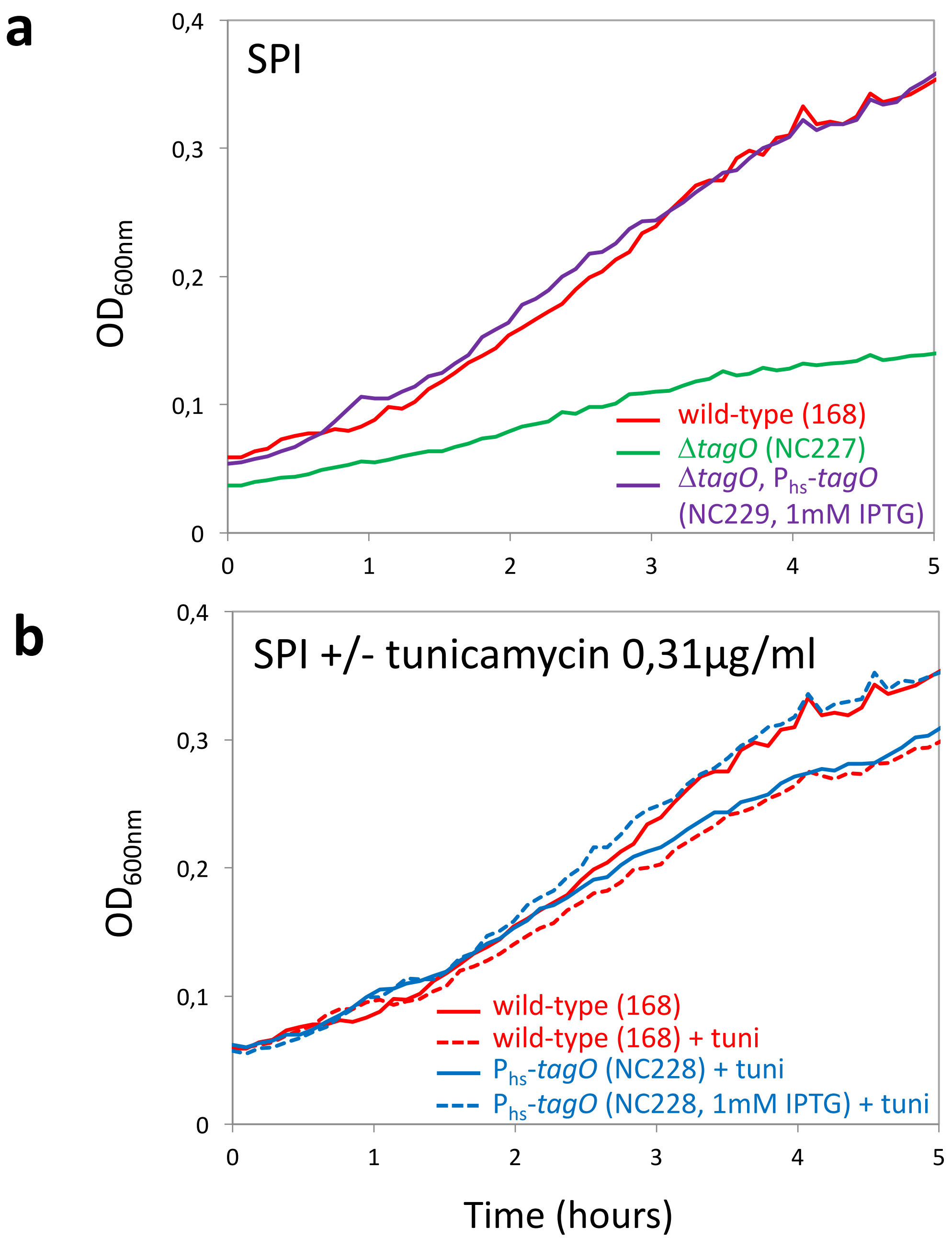

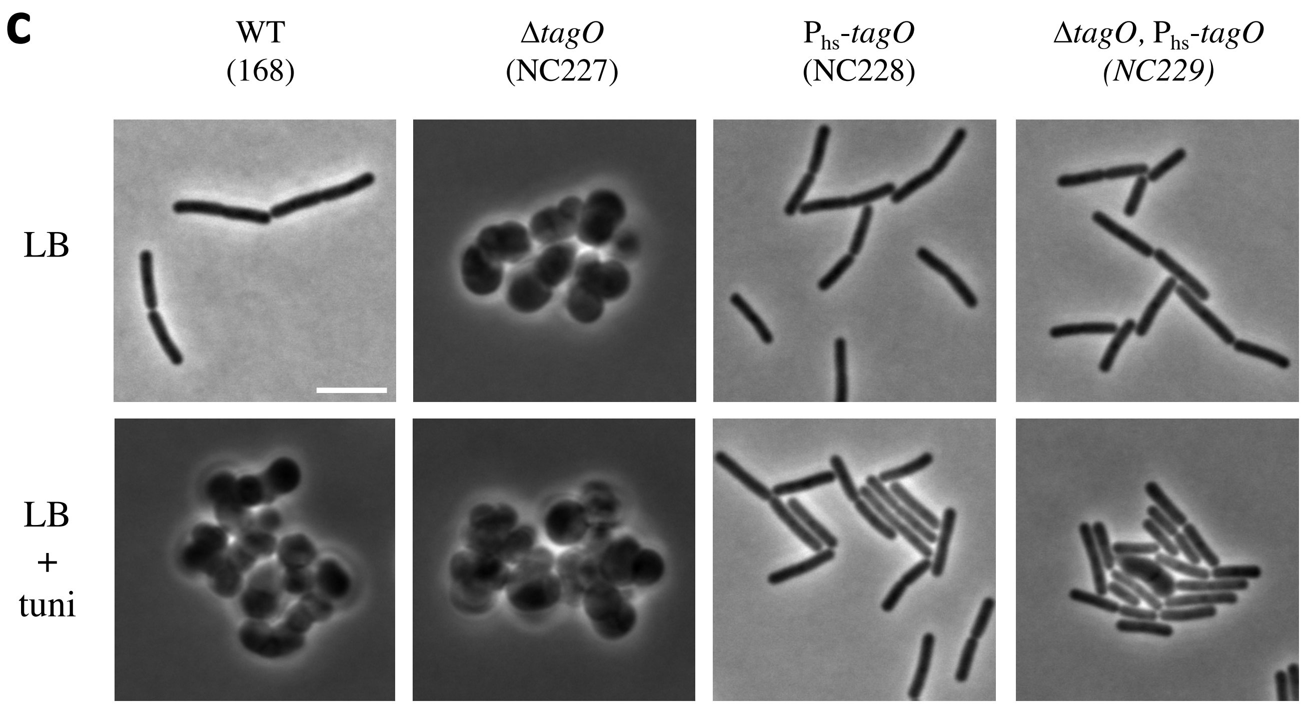

Supplementary Fig. 3. Functionality of P_{hs}-*tagO*.

a-b. Growth of strains 168 (wild-type, red), NC227 (Δ*tagO*, green), NC228 (P_{hs}-*tagO*, blue) and NC229 (Δ*tagO* P_{hs}-*tagO*, purple) was monitored in SPI (a) and in SPI supplemented with 0.31 µg/ml tunicamycin (b). Expression of *tagO* from the hyperspank promoter (Phs) was induced using 1mM IPTG with strain NC228 and NC229.

c. Representative phase contrast images of cells of various genetic backgrounds (WT, strain 168; Δ*tagO*, strain NC227; P_{hs}-*tagO*, strain NC228; Δ*tagO* P_{hs}-*tagO*, strain NC229) exponentially growing in LB (top panels) or in LB supplemented with tunicamycin (0.31µg/ml, bottom panels). Complementary data to Fig. 2a. Scale bar, 5µm.

Supplementary Fig. 4

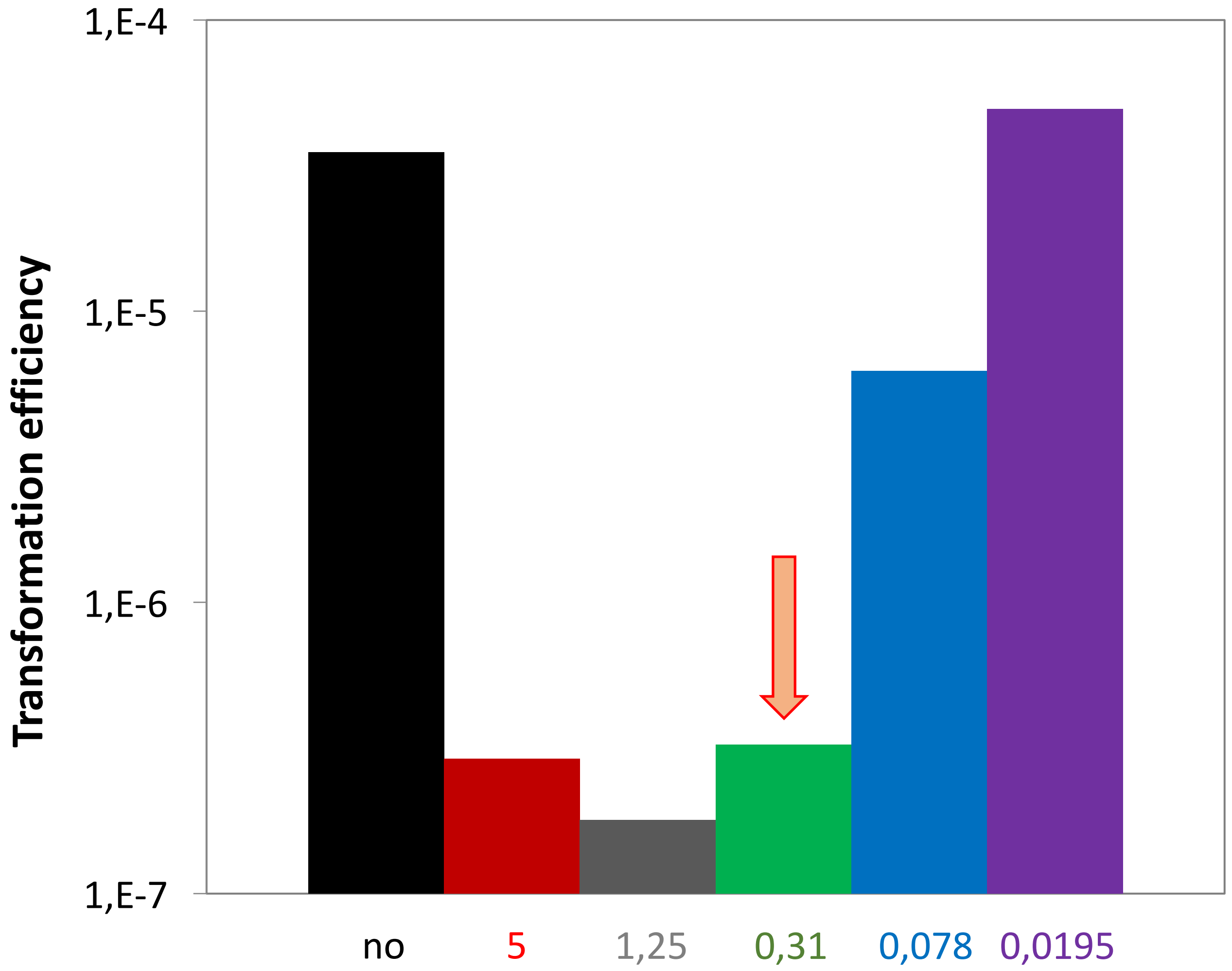

Supplementary Fig. 4. Determination of the lowest tunicamycin concentration providing full inhibition of genetic transformation.

In order to optimize our chances to complement the effect of tunicamycin on genetic transformation by over-expressing the target of tunicamycin, *tagO*, we first determined the lowest antibiotic concentration providing full inhibition of genetic transformation. The effect of serial 4-fold tunicamycin dilutions (5, 1.25, 0.31, 0.078 and 0.0195 μg/ml) on the transformation efficiency of the wild-type strain (168) was tested using the two-steps protocol. 0,31μg/ml of tunicamycin (arrow) was the lowest concentration showing a maximum inhibition of genetic transformation.

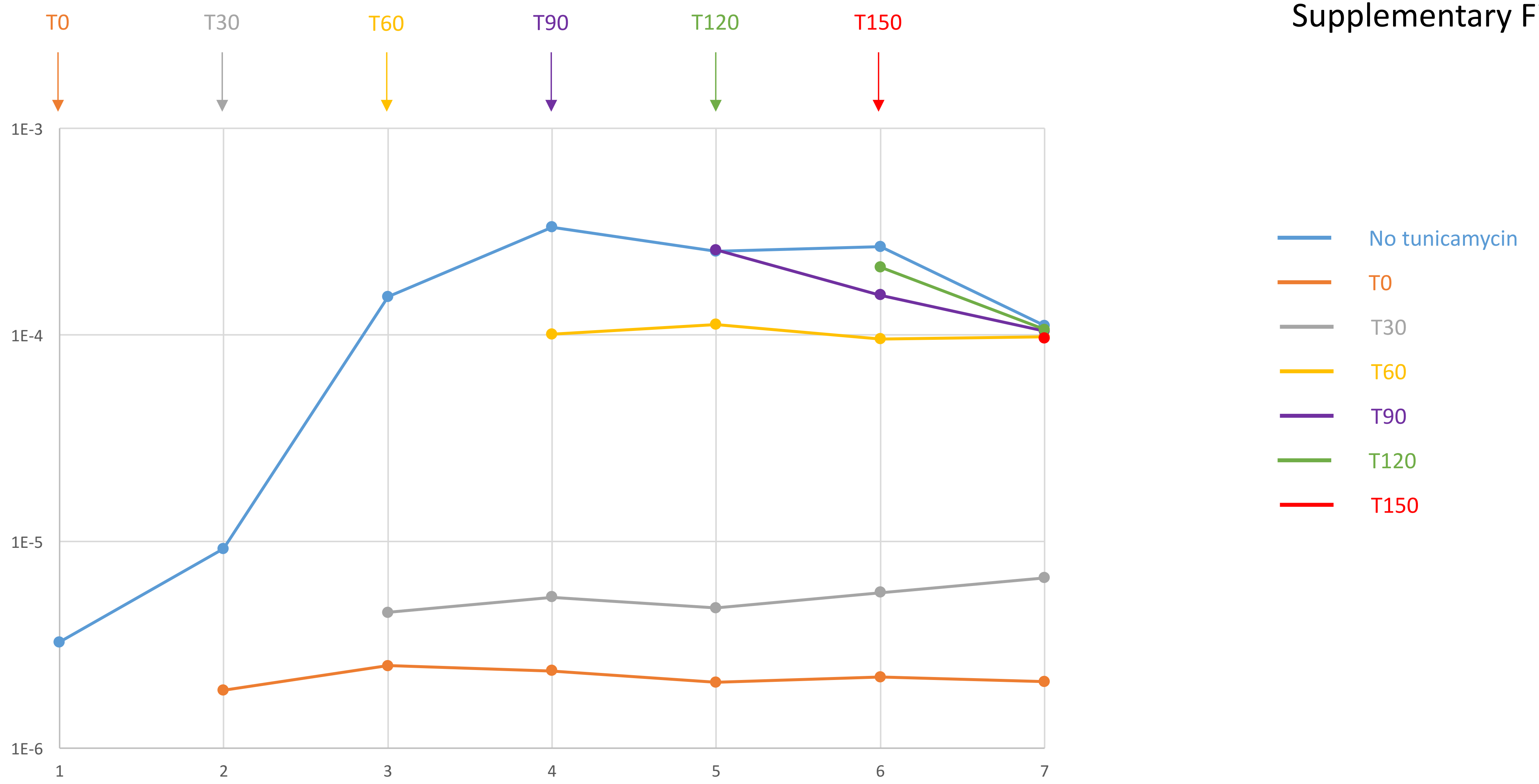

Supplementary Fig. 5. Time series experiment show that active WTA synthesis during competence is required for efficient genetic transformation.

For details of the experimental procedure, see Supplementary Fig. 1. When wild-type strain 168 *B. subtilis* cells reached stationary phase in SPI medium (T_0 in Fig. 1 and Supplementary Fig. 1), the culture was diluted in SPII medium. Tunicamycin (5 μg/ml) was added at the indicated time points (colored arrows). For each time point, viability and the number of transformants per ml were evaluated by plating on the adequate selective media every 30 min after addition of the drug. Transformation efficiencies were calculated by dividing the number of transformants/ml by the viability at each time point. The color of each curve corresponds to the color of the arrow indicating the time of tunicamycin addition.

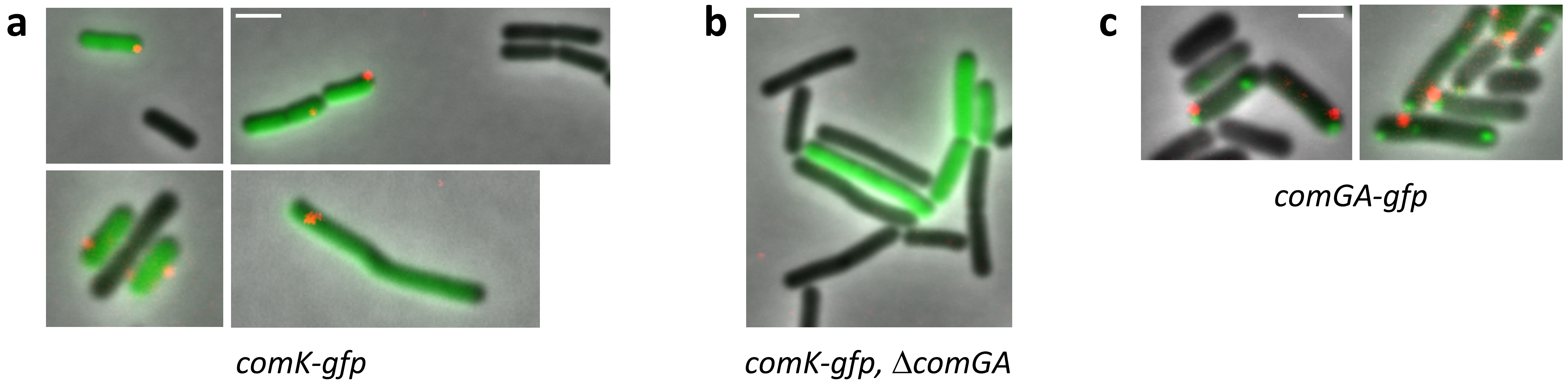

Supplementary Fig. 6. Visualization of fluorescently-labelled DNA at the surface of competent cells.

To confirm that binding of fluorescently-labelled DNA was specific and dependent on competence development, we conducted the following experiments. Strains NC59 (a), NC164 (b) and NC58 (c), all three of which develop competence comparably to the wild-type strain (~ 5%), were grown to stationary phase in SPI medium and diluted in fresh SPII medium. After 120 minutes of growth in SPII medium, fluorescently-labelled DNA (ATTO550-dUTP, 2μg/ml) was added for 8 minutes. Cells were washed twice in fresh SPII medium and observed under the microscope. Scale bar, 2µm.

a. Visualization of fluorescently-labeled DNA at the surface of NC59 cells (*comK-gfp*). The presence of the *comK-gfp* fusion expressed under the control of the *comK* promoter allows to differentiate competent from non-competent cells. Bound DNA forms foci at the surface of competent cells.

b. Visualization of fluorescently-labeled DNA at the surface of NC164 cells (*comk-gfp*, Δ*comGA*). Because ComGA is essential for exogenous DNA binding at the surface of the competent cells [6], no DNA binding can be observed.

c. Visualization of fluorescently-labeled DNA at the surface of NC58 cells (*comGA-gfp*). Competent cells can be distinguished by the presence of ComGA-GFP foci near the poles and throughout the cell [7]. Bound DNA can be seen as foci on the surface of the cells, most of the time in the vicinity of ComGA clusters.

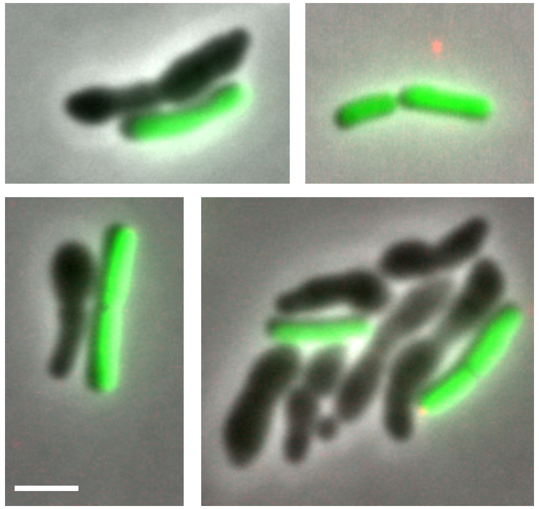

Supplementary Fig. 7. Binding of fluorescently-labelled DNA at the surface of tunicamycin-treated cells.

Examples of tunicamycin-treated wild-type cells natively expressing *comK-gfp* as a competence reporter (strain NC59). Only non competent cells display abnormal morphologies. DNA binding is almost completely lost at the surface of competent cells. Complementary images to Fig. 3c. Scale bar, 2μm.

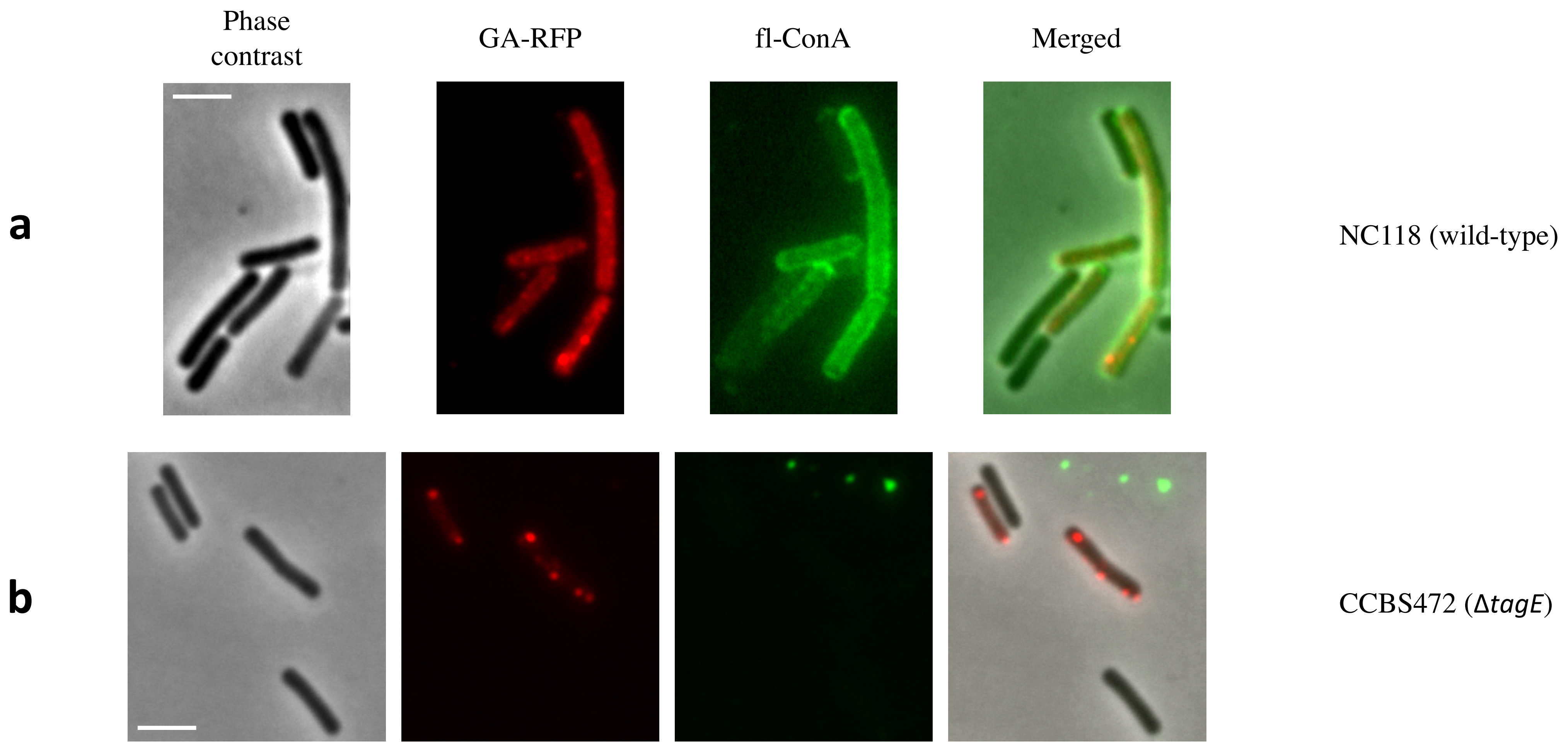

Supplementary Fig. 8. Fl-ConA specifically stains glucosylated WTA.

Comparison of NC118 (*comGA-mrfpruby*, mcComS. Panel a) and CCBS472 (*comGA-mrfpruby*, Δ*tagE*, mcComS. Panel b) cells stained with fl-ConA reveals that ConA specifically labels glucose residues decorating WTA. Scale bar, 2μm.

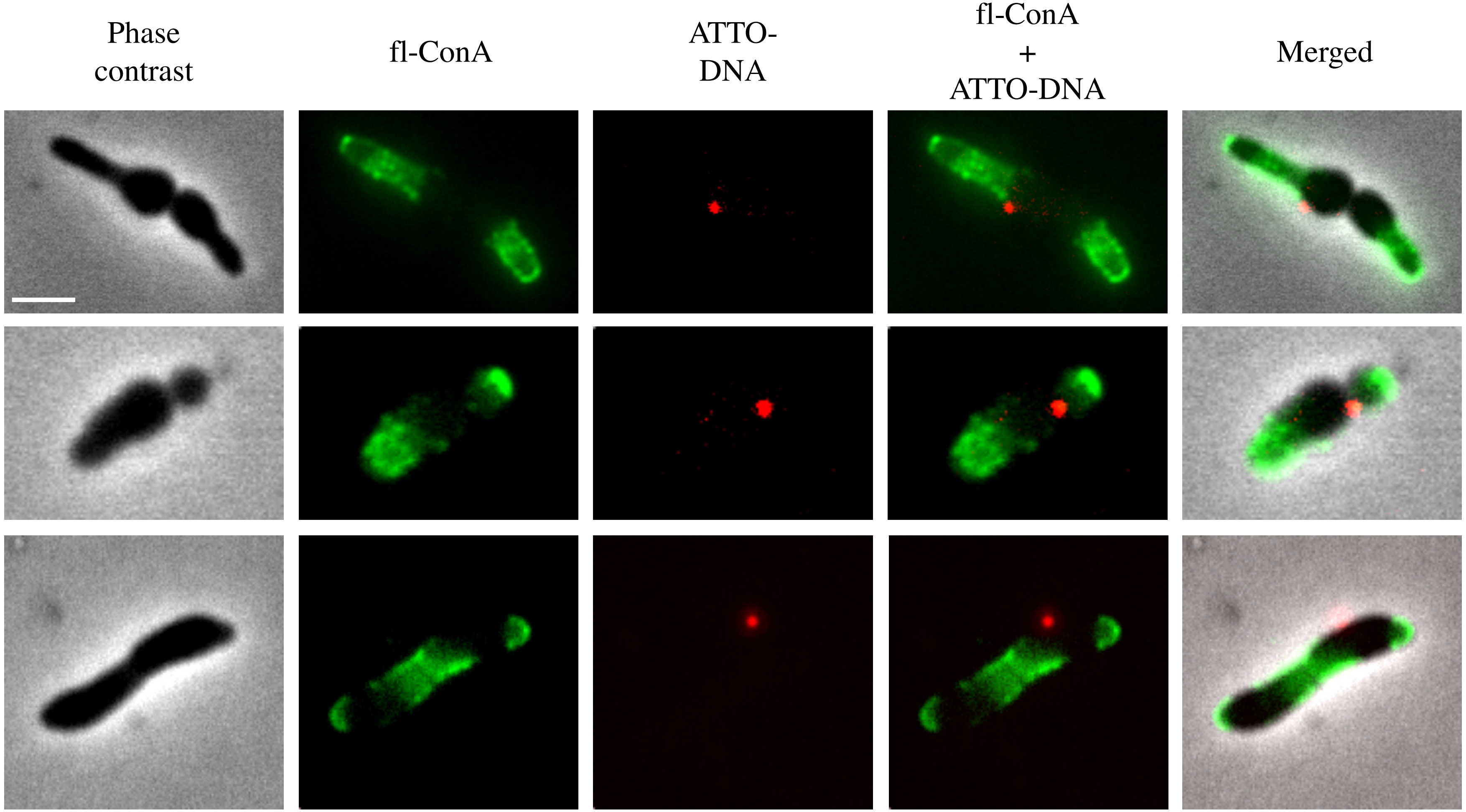

Supplementary Fig. 9. Exogenous DNA binds to WTAs-depleted regions at the surface of non competent tunicamycin-treated cells.

Representative cells of the wild-type strain (168) grown for 2h in SPII medium supplemented with tunicamycin (5µg/ml) prior to exposure to fluorescently-labelled DNA (ATTO550-dUTP, 2µg/ml) for 8 min followed by fl-ConA labelling for 10 min. Scale bar, 2µm.

Supplementary Fig. 10

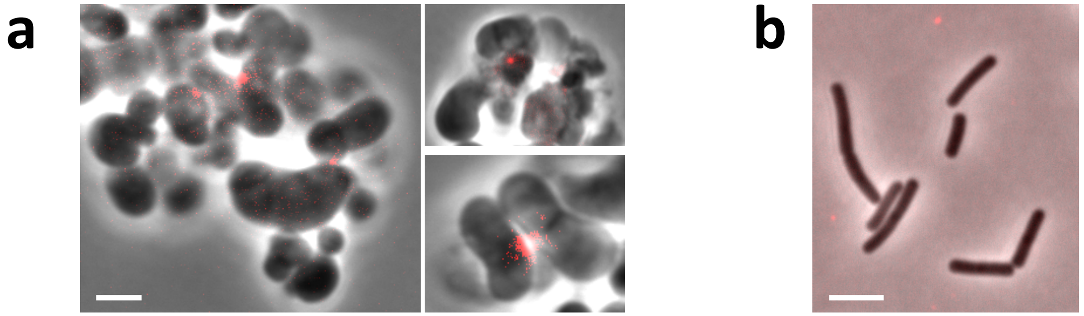

Supplementary Fig. 10. DNA binding at the surface of *tagO* mutant cells.

ΔtagO mutant cells (NC227) (a) and wild-type cells (168) (b) were grown in LB to exponential phase and exposed to fluorescently-labelled DNA (ATTO550-dUTP, 2μg/ml) for 8 minutes. Cells were then washed in fresh SPII medium and observed under the microscope. Because of the abnormal round shape of Δ*tagO* mutant cells and their tendency to aggregate, counting the percentage of cells binding DNA was not possible. Scale bar, 2μm.

Supplementary Fig. 11

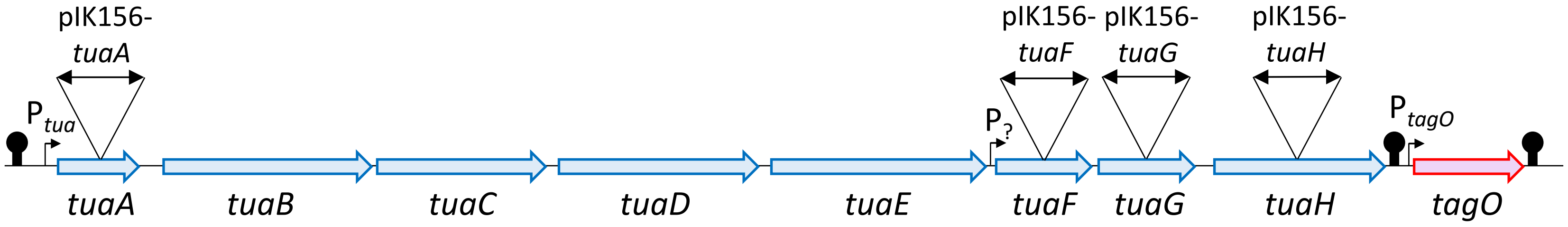

Supplementary Fig. 11. Organization of the *tua* operon in *B. subtilis*.

The eight genes of the *tua* operon are presented (*tuaA-H*). The *tagO* gene is found directly downstream the *tua* operon. Known promoters are annotated in front of the *tua* operon (P_{tua}) and the *tagO* gene (P_{tagO}). The putative, competence-induced internal promoter upstream *tuaF* is also shown ($P_{?}$). Terminator sequences are indicated with black circles. Double-headed arrows illustrate the place of insertion of the gene-disruption plasmids (pIK156::*tuaA*, ::*tuaF*, ::*tuaG* and ::*tuaH*).

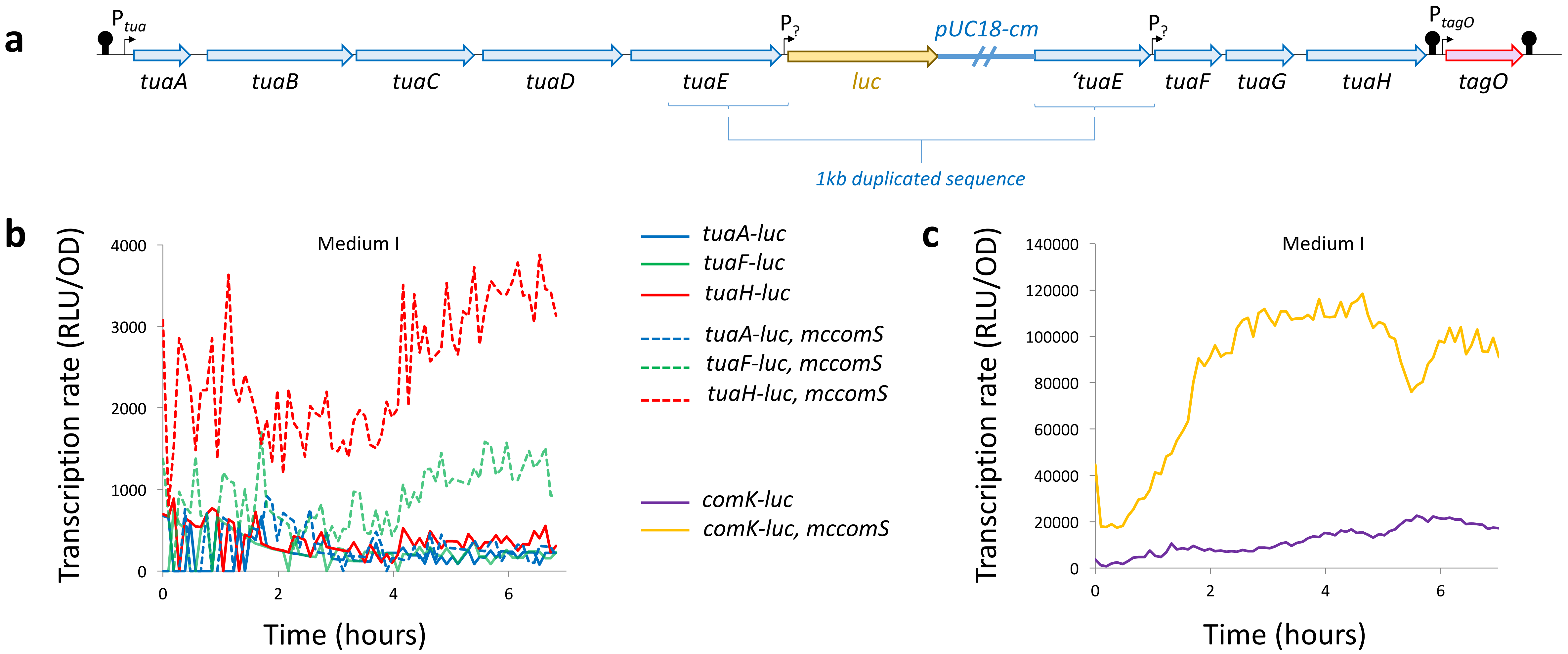

Supplementary Fig. 12. ***tuaF*, *G* and *H* are overexpressed during competence.**

a. Example of insertion for the pUC18cm-*tuaF-luc* at the native locus. Briefly, a 1 kbp fragment present directly upstream of *tuaF* was cloned in front of the luciferase gene in the pUC18cm plasmid. This plasmid was then inserted at the native locus by single cross-over placing the luciferase gene under the control of all the transcriptional regulatory sequences upstream of *tuaF* on the chromosome. This insertion results in the duplication of the 1 kbp fragment downstream of the plasmid. The same kind of construct was inserted in front of *tuaA* and *tuaH* genes.

b. Expression profiles in SPI for *tuaA (blue curves), tuaF (green curves) and tuaH (red curves)* in the wild-type (plain curves) and in the mccomS (dotted curves) backgrounds.

c. Control expression profiles in SPI for *comK* in the wild-type (purple curve) and the mccomS (orange curve) backgrounds. When normal regulatory pathways are bypassed (mccomS background), competence is induced in nearly 60% of the population, even in medium SPI where very few transformants are detected in a wild-type background.

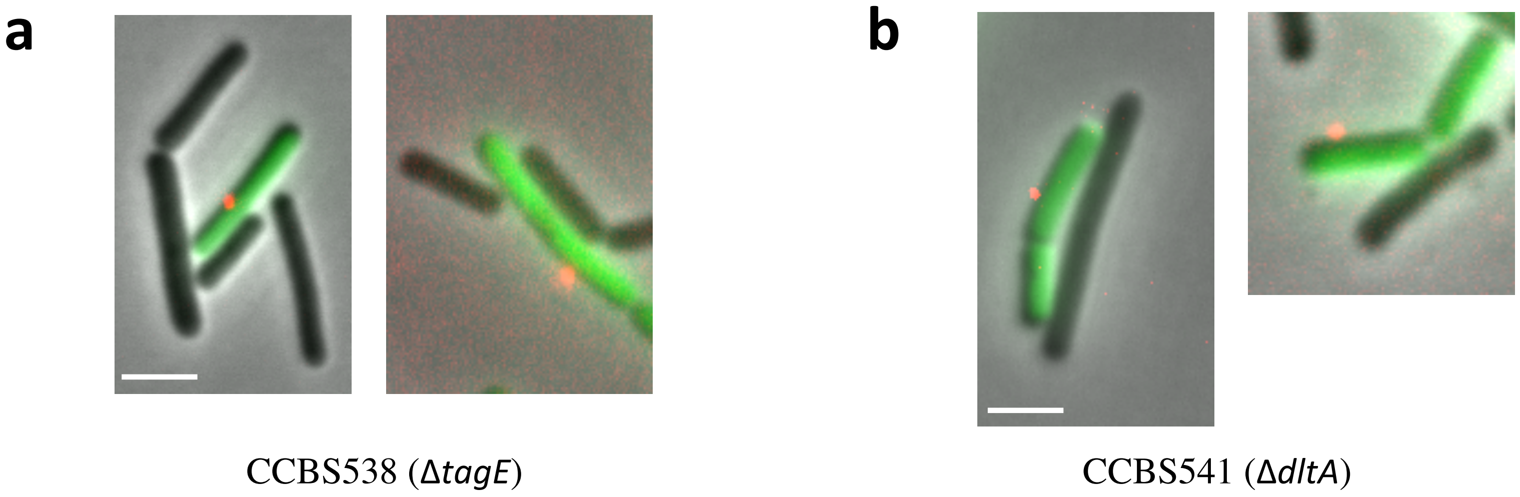

Supplementary Fig. 13. ***Deletion of tagE, dltA does not affect DNA binding at the surface of competent cells.***

Examples of competent cells (expression ComK-GFP) binding fluorescently-labeled DNA (ATTO550-dUTP) in Δ*tagE* (CCBS538, a) and Δ*dltA* (CCBS541, b) mutant backgrounds.

Scale bar, 2μm.

SUPPORTING TABLES

Supplementary Table 1. ***B. subtilis*** **trains.**

Strain	Genotype	Source (1)
168	trpC2	[1]
NC3	trpC2 P_{comGA}-*luc* (Cm)	This study
NC58	trpC2 amyE::P_{comGA}-*comGA-gfp* (Cm)	[2]
NC59	trpC2 P_{comK}-*comK-gfp* (Cm)	[3]
NC118	trpC2 *thrC*::P_{comGA}-*comGA-mrfpruby* (Ery) mcComS (Kan)	[3]
NC164	trpC2 P_{comK}-*comK-gfp* (Cm) *comGA*::*Tn917* (Ery)	This study
NC227	trpC2 *tagO*::Ery (Ery)	This study
NC228	trpC2 *amyE*::$P_{hyperspank}$-*tagO* (Kan)	This study
NC229	trpC2 *tagO*::Ery (Ery) *amyE*::$P_{hyperspank}$-*tagO* (Kan)	This study
NC230	trpC2 *tagO*::Ery (Ery) P_{comK}-*comK-gfp* (Cm)	This study
NC238	trpC2 *tuaA*::pIK156-A (Spc)	This study
NC239	trpC2 *tuaF*::pIK156-F (Spc)	This study
NC240	trpC2 *tuaG*::pIK156-G (Spc)	This study
NC241	trpC2 *tuaH*::pIK156-H (Spc)	This study
NC243	trpC2 *tuaH*::pIK156-H (Spc) *amyE*::$P_{hyperspank}$-*tagO* (Kan)	This study
NC275	trpC2 *tuaH*::pIK156-H (Spc) P_{comK}-*comK-gfp* (Cm)	This study
NC281	trpC2 *tuaA*::pIK156-A (Spc) *amyE*::$P_{hyperspank}$-*tuaH* (Kan)	This study
NC282	trpC2 *tuaF*::pIK156-F (Spc) *amyE*::$P_{hyperspank}$- *tuaH* (Kan)	This study
NC283	trpC2 *tuaG*::pIK156-G (Spc) *amyE*::$P_{hyperspank}$- *tuaH* (Kan)	This study
NC284	trpC2 *tuaH*::pIK156-H (Spc) *amyE*::$P_{hyperspank}$- *tuaH* (Kan)	This study
NC288	trpC2 $\Delta tagGH^{Bs}$ (Cm) *amyE*::$P_{hyperspank}$- $tarGH^{Sa}$ (Spc)	This study
CCBS487	trpC2 Δ*dltA* (Kan)	This study
CCBS536	trpC2 Δ*tagE* (Spc)	This study
CCBS503	trpC2 P_{tuaA}-*luc* (Cm)	This study
CCBS504	trpC2 P_{tuaF}-*luc* (Cm)	This study
CCBS505	trpC2 P_{tuaH}-*luc* (Cm)	This study
CCBS506	trpC2 P_{tuaA}-*luc* (Cm) mcComS (Kan)	This study
CCBS507	trpC2 P_{tuaF}-*luc* (Cm) mcComS (Kan)	This study
CCBS508	trpC2 P_{tuaH}-*luc* (Cm) mcComS (Kan)	This study
NC129	trpC2 P_{comK}-*luc* (Cm)	[3]
CCBS546	trpC2 P_{comK}-*luc* (Cm) mcComS (Kan)	This study
CCBS472	trpC2 Δ*tagE* (Spc) *thrC*::P_{comGA}-*comGA-mrfpruby* (Ery) mcComS (Kan)	This study
CCBS538	trpC2 Δ*tagE* (Spc) P_{comK}-*comK-gfp* (Cm)	This study
CCBS541	trpC2 Δ*dltA* (Kan) P_{comK}-*comK-gfp* (Cm)	This study

(1) The source of the constructs and strains not generated in this study is as follow:

The P_{comK}-*luc* and P_{comGA}-*luc* constructs come from strains BD4773 [4] and BD4880 respectively, constructed in D. Dubnau's lab. The mcComS and P_{comK}-*comK-gfp* constructs come from strain BD4015 [5] generated in D. Dubnau's lab. The amyE::P_{comGA}-*comGA-gfp* construct comes from strain BD3773 [2] realized in D. Dubnau's lab. The *tagO* knock out comes from strain EB1451 from the Brown lab [6]. The *comGA* knock out comes from strain BD1248 realized in D. Dubnau's lab [7]. The $tagGH^{Bs}$ knock out and *amyE*::$P_{hyperspank}$- $tarGH^{Sa}$ construct come from strain KS002 realized in S. Walker's lab [8]. The *tagE* knock out was a kind gift from Eric Brown [9]. The *dltA* and *yfhO* knock out strains came from the *Bacillus subtilis* stock center.

References

1. Albertini, A. M. & Galizzi, A. The sequence of the trp operon of Bacillus subtilis 168 (trpC2) revisited. *Microbiology* **145 (Pt 1,** 3319–3320 (1999).

2. Maier, B., Chen, I., Dubnau, D. & Sheetz, M. P. DNA transport into Bacillus subtilis requires proton motive force to generate large molecular forces. *Nat. Struct. Mol. Biol.* **11,** 643–649 (2004).

3. Mirouze, N., Ferret, C., Yao, Z., Chastanet, A. & Carballido-López, R. MreB-Dependent Inhibition of Cell Elongation during the Escape from Competence in Bacillus subtilis. *PLoS Genet.* **11,** (2015).

4. Mirouze, N., Desai, Y., Raj, A. & Dubnau, D. Spo0A~p imposes a temporal gate for the bimodal expression of competence in bacillus subtilis. *PLoS Genet.* **8,** (2012).

5. Maamar, H. & Dubnau, D. Bistability in the Bacillus subtilis K-state (competence) system requires a positive feedback loop. *Mol. Microbiol.* **56,** 615–624 (2005).

6. D'Elia, M. A., Millar, K. E., Beveridge, T. J. & Brown, E. D. Wall teichoic acid polymers are dispensable for cell viability in Bacillus subtilis. *J Bacteriol* **188,** 8313–8316 (2006).

7. Hahn, J., Albano, M. & Dubnau, D. Isolation and characterization of Tn917lac-generated competence mutants of Bacillus subtilis. *J Bacteriol* **169,** 3104–3109 (1987).

8. Schirner, K., Stone, L. K. & Walker, S. ABC transporters required for export of wall teichoic acids do not discriminate between different main chain polymers. *ACS Chem Biol* **6,** 407–412 (2011).

9. Allison, S. E., D'Elia, M. A., Arar, S., Monteiro, M. A. & Brown, E. D. Studies of the genetics, function, and kinetic mechanism of TagE, the wall teichoic acid glycosyltransferase in Bacillus subtilis 168. *J. Biol. Chem.* **286,** 23708–23716 (2011).

Supplementary Table 2. Primers used in this study.

Name	Sequence
amyE*::P$_{hyperspank}$-*tagO	
amy-F	5'- CCCGACATCCGGCGTTCTCATGGC -3'
amy-R	5'- TCAATGGGGAAGAGAACCGCTTAAGCCC -3'
pDP111-MCS-F	5'- GGCTAGCTGTCGACTAAGCTTAATTGTTATCCGC -3'
pDP111-MCS-R	5'- GCATGCAAGCTAATTCGGTGGAAACGAGG -3'
MCS-RBS-tagO-F	5'-GCGGATAACAATTAAGCTTAGTCGACAGCTAGCCACTAAG GAGAAGGTATA TGGAAGGAACGGTTCTC -3'
MCS-tagO-R	5'-CCTCGTTTCCACCGAATTAGCTTGCATGCTTAATTCCTTTT CACCAGCCGTTTATAAAACTTTGTAAACGG C -3'
amyE*::P$_{hyperspank}$-*tuaH	
amy-F	5'- CCCGACATCCGGCGTTCTCATGGC -3'
amy-R	5'- TCAATGGGGAAGAGAACCGCTTAAGCCC -3'
pDP111-MCS-F	5'- GGCTAGCTGTCGACTAAGCTTAATTGTTATCCGC -3'
pDP111-MCS-R	5'- GCATGCAAGCTAATTCGGTGGAAACGAGG -3'
MCS-RBS-tuaH-F	5'-GCGGATAACAATTAAGCTTAGTCGACAGCTAGCCGCTGCA AACAGAGAGAAAAGGTGATGAAGGTGG-3'
MCS-tuaH-R	5'- CCTCGTTTCCACCGAATTAGCTTGCATGCCGCATGTTTTTG AATCCCTGTATATTTCAT-3'
**Gene deletion**	
sacI-tuaA-F	5'-GCAGCAGAGCTCGTGAGTGCAGAGAAAAGCATGAATGTGAG -3'
bamH1-tuaA-R	5'-GCAGCAGGATCCAATATCAGCTTTTCCTTCGGCGTCATATCG-3'

sacI-tuaF-F	5'-GCAGCAGAGCTCATGAACGATATATTGATAAGAATAGCCCGTC-3'
bamH1-tuaF-R	5'-GCAGCAGGATCCAAAGGATTCTCTGAAAAATTCAG GAATGACAA-3'
sacI-tuaG-F	5'-GCAGCAGAGCTCATGACCAATTGGAAACCTCTCGTATCT-3'
bamH1-tuaG-R	5'- GCAGCAGGATCCTAATCTTTTTTTCACCGCGTTTTT TGCATATTG-3'
sacI-tuaH-F	5'- GCAGCAGAGCTCCGTTGCCACTGCTGAATGGGGAAAAGA -3'
bamH1-tuaH-R	5'- GCAGCAGGATCCTCCAATCTTGTGTCCGGGCGATGTTTC -3'

Luciferase transcriptional fusions

382-PtuaA_PtsI_fw	5'- AATCCTGCAGCTTGCTGCAAATATCGTACAAA -3'
383-PtuaA_KpnI_rev	5'- AATCGGTACCTGGGATGTTATGATGACCTATGC -3'
384-PtuaF_PtsI_fw	5'- AATCCTGCAGTTGGCAATGGGGATCTTCTTCA -3'
385-PtuaF_KpnI_rev	5'- AATCGGTACCTAGTCTGTTTATCTGTACATCGG -3'
386-PtuaH_PtsI_fw	5'- AATCCTGCAGCGAGTCGGTGAGCGAGGATTC -3'
387-PtuaH_KpnI_rev	5'- AATCGGTACCCTCTGTTTGCAGCTATAATCTT -3'
